# Multi-Stage Treatment for Spetzler–Martin Grades III, IV, and V Arteriovenous Malformations: Preoperative Embolization and Microsurgical Resection in a Consecutive Series of 250 Patients

**DOI:** 10.3390/jcm12185990

**Published:** 2023-09-15

**Authors:** Marcel Alfter, Pablo Albiña-Palmarola, Alexandru Cimpoca, Roberto Díaz-Peregrino, Paul Jans, Oliver Ganslandt, Dietmar Kühne, Hans Henkes

**Affiliations:** 1 Neuroradiological Clinic, Klinikum Stuttgart, 70174 Stuttgart, Germany; pablo.a.med@gmail.com (P.A.-P.); hhhenkes@aol.com (H.H.); 2 Medical Faculty, University Duisburg-Essen, 47057 Duisburg, Germany; 3 Department of Anatomy, School of Medicine, Pontificia Universidad Católica de Chile, Santiago 8331150, Chile; 4 Department of Neurosurgery, University Hospital Heidelberg, Ruprecht-Karls-University Heidelberg, 69117 Heidelberg, Germany; 5 Clinic for Neurosurgery, Alfried Krupp Krankenhaus, 45131 Essen, Germany; 6 Neurosurgical Clinic, Klinikum Stuttgart, 70174 Stuttgart, Germany; 7 Clinic for Radiology and Neuroradiology, Alfried Krupp Krankenhaus, 45131 Essen, Germany

**Keywords:** arteriovenous malformation, embolization, multimodal treatment, microsurgical resection

## Abstract

Purpose. The treatment of high-grade brain AVMs is challenging and has no guidelines available to date. This study was aimed at reporting the experience of two centers in treating these AVMs through a multi-stage approach consisting of preoperative embolization and microsurgical resection. Methods. A retrospective review was performed for 250 consecutive patients with a diagnosis of high-grade brain AVM (Spetzler–Martin grades III, IV, and V) treated in two centers in Germany between January 1989 and February 2023. The analyzed data included demographic, clinical, morphological, and neurological data. Results. A total of 150 cases (60%) were classified as Spetzler–Martin grade III, 82 cases (32.8%) were classified as grade IV, and 18 cases (7.2%) were classified as grade V. Eighty-seven cases (34.8%) presented with hemorrhage. The devascularization percentages achieved were <50% in 24 (9.6%), 50–80% in 55 (22%), and >80% in 171 (68.4%) cases. The average number of sessions was 5.65 ± 5.50 and 1.11 ± 0.32 endovascular and surgical procedures, respectively, and did not significantly differ by rupture status. Death or dependency (mRS score ≥ 3) after the last follow-up was observed in 18.8% of patients and was significantly associated with age > 80 years and poor baseline neurological condition. The complete resection rate was 82.3% and was significantly associated with age > 80 years, large nidus, and deep venous drainage. Permanent disabling neurological deficit after at least 3 months of follow-up was diagnosed in 13.2% of patients and was significantly associated with age > 80 years and infratentorial locations. Conclusion. A multi-stage treatment for high-grade AVMs is feasible for selected cases but comes at a functional cost. The devascularization percentage was not associated with the investigated outcomes. Age > 80 years was associated with poor safety and effectiveness outcomes; consequently, this treatment should be offered only in exceptional circumstances.

## 1. Introduction

Brain arteriovenous malformations (AVMs) are uncommon vascular lesions with presenting symptoms including intracranial hemorrhage (ICH), seizures, progressive neurological deficits, and headaches. With the widespread use of MRI, AVMs are increasingly being diagnosed in asymptomatic patients [1]. Despite advances in neuroradiology, vascular neurosurgery, and radiotherapy, these lesions continue to be considered a multidisciplinary challenge and to pose potentially devastating risks to patients.

The decision to treat remains controversial and depends on several factors, including the balance between the risks associated with the natural history of the lesion, primarily hemorrhagic risk, and those associated with their treatment. Several questions remain to be answered regarding the natural history of unruptured AVMs, a fact highlighted by the intense backlash from the neurosurgical and neurointerventional communities to the ARUBA trial [2,3,4], the first prospective randomized trial comparing medical management with interventional treatment for unruptured AVMs [5]. Recently, the potentially damaging effect of the shift in favor of non-interventional management generated by the trial mentioned above has been published, suggesting that withholding of treatment in patients for whom intervention was indicated might have contributed to the increased incidence of AVM ruptures and in-hospital mortality observed across hospitals in the United States [6].

Several demographic, morphological, and angioarchitectural risk factors for hemorrhage have been described [7], and higher hemorrhagic risk has also been reported for high-grade AVMs [8,9], thus further complicating the decision-making process. In any case, the goal of any intervention is to eliminate precisely this risk, which is only accomplished after the complete exclusion of the lesion. Different treatment options exist, including microsurgical resection, endovascular embolization, stereotactic radiosurgery (SRS), and combined multimodal treatment. Conservative management is indicated when a cure is not feasible or the associated risk of therapy is considered unacceptable.

Microsurgical resection is still considered the gold standard for the treatment of low-grade AVMs because it achieves excellent curative results with relatively low complication rates; however, the perioperative risks are significantly elevated for grades III, IV, and V AVMs [10,11]. SM grade III AVMs are a heterogeneous group that can encompass four combinations of size, venous drainage, and eloquence. According to Spetzler et al., these lesions lie at the limit of operability and are usually managed through multimodal approaches [12]. Furthermore, SM grade IV and V AVMs have been proposed to have better outcomes when treated conservatively [13], and some researchers have considered that the risk makes surgery prohibitive [12]. Treatment of these lesions is usually reserved for exceptional cases, such as those with recurrent bleeding, progressive neurological deficit, and high-risk features.

Endovascular preoperative embolization has been proposed as an advantageous complement to surgery for large and complex AVMs because of its potential to eliminate deep arterial feeders that usually limit resectability. In addition, this treatment has been found to significantly decrease arterial inflow to the nidus, thereby diminishing intraoperative blood loss and favoring effective dissection from the surrounding cortical areas [14,15]. Preoperative embolization can be used either as a multi-stage progressive devascularization strategy or as part of single-stage hybrid procedures. Advocates of multi-stage treatments have proposed that progressively decreasing the blood flow into the nidus through subsequent embolization sessions may decrease the risk of normal perfusion pressure breakthrough, which is considered the main cause of postoperative hemorrhage in high-flow and high-volume AVMs [16,17]. In contrast, researchers proposing novel hybrid single-stage schemes have highlighted the potential drawbacks of the additive procedural risk with each endovascular session [18].

The effects of multimodal treatment consisting of preoperative embolization and subsequent surgery, compared with those of stand-alone microsurgical resection, are unclear [19]. Uncertainty is particularly high for the subgroup of patients with high-grade AVMs because only a few small studies in highly selected patients have been published [18,20]. Distinguishing which patients would benefit most from treatment and which patients should be managed conservatively is essential. Therefore, the goal of this study was to present our experience spanning three decades of treatment of high-grade AVMs by using a multi-stage approach consisting of preoperative embolization and subsequent microsurgical resection, and to investigate factors potentially associated with safety and effectiveness outcomes. To our knowledge, this is the largest published series on this topic in this subgroup of lesions.

## 2. Materials and Methods

### 2.1. Study Design

Between January 1989 and February 2023, 250 consecutive patients with a diagnosis of high-grade brain AVMs (SM grades III, IV, and V) underwent preoperative embolization and microsurgical resection at two German centers, *Alfried Krupp Krankenhaus* in Essen (from 1983 to 2006) and *Katharinenhospital* in Stuttgart (from 2007 to 2023). The information was retrospectively collected and analyzed until 2015 and then prospectively. Some patients treated at *Alfried Krupp Krankenhaus* before 1998 have been discussed in a previous article [21]; however, they were included in this study to provide updated follow-up. The information was obtained from the electronic databases and archives from both centers, and long-term follow-up data were gathered in person or through structured telephone evaluations of the patients or their family members by a neurologist, neuroradiologist, or stroke study nurse.

All included patients had a diagnosed brain AVM confirmed by digital subtraction angiography (DSA) and received multimodal treatment consisting of at least one session of preoperative embolization followed by microsurgical resection. Patients diagnosed with dural arteriovenous fistulas, spinal or facial AVMs, vein of Galen malformations, or more than two AVMs were excluded from this study. For patients with two AVMs, each lesion was considered and analyzed separately.

### 2.2. Data Collection

The baseline characteristics collected included demographic data, clinical presentation, SM grade of the AVM [10], lesion size, localization (supratentorial, infratentorial, or midline), side, eloquence (sensorimotor cortex, visual cortex, basal ganglia including the internal capsule, thalamus, hypothalamus, cerebellar peduncles, cerebellar nuclei, and brainstem), presence of deep venous drainage (with or without superficial venous drainage), rupture status, type of hemorrhage (classified as intracerebral, subarachnoid, or intraventricular hemorrhage), presence of aneurysms, aneurysm type (flow-related or intranidal), and presence of venous varices. Radiological information was retrieved from the records originally described by attending neuroradiologists from both centers. Attending neurologists and neurosurgeons classified the functional neurological status of each case according to the seven-step modified Rankin Scale (mRS) score [22]. The scores and details regarding neurological complications were obtained at the time of presentation, after preoperative embolization, after surgery, and after the last follow-up.

The treatment strategy was decided for each patient in a multidisciplinary neurovascular meeting among interventional neuroradiologists, neurologists, and vascular neurosurgeons, according to each case’s clinical and radiological features. Written informed consent was obtained from all patients or their legal caregivers before the start of treatment, after the treating specialists had thoroughly explained the risks and benefits. Ethics approval for the retrospective data analysis was obtained from the local ethics committee (Ethik-Kommission bei der Landesärztekammer Baden Württemberg, F-2018-080).

### 2.3. Endovascular Treatment

All angiographic evaluations and embolization procedures were performed by experienced interventional neuroradiologists using digital subtraction technology. Until 1991, a first-generation DSA unit (DVI, Philips, Eindhoven, The Netherlands) was used in Essen. In 1990, a monoplane DSA unit was added (Angiostar, Siemens, Erlangen, Germany). In 1992, a biplane system was introduced and has since been used for most procedures (Neurostar, Siemens, Erlangen, Germany). In Stuttgart, interventions were performed on biplane DSA systems (Axiom Artis, Siemens, Erlangen, Germany; Allura, Philips, Eindhoven, The Netherlands). The embolization procedures were performed in patients under conscious sedation or general anesthesia in both Stuttgart and Essen. Anticoagulation was achieved with a 3000–5000 IU bolus dose of unfractionated heparin at the start of the procedure and subsequent 1000 IU bolus doses every hour to maintain an activated clotting time of 2–2.5 times baseline. No heparin was administered in the acute phase of intracranial hemorrhage. Standard biplane DSA runs were acquired and used to select target vessels for the embolization procedure. For embolization of the AVM feeders, wire-guided microcatheters (e.g., Tracker-18 Unibody, Target Therapeutics; Marathon, Medtronic) and flow-guided microcatheters (Magic 1.2, Balt Extrusion, Montmorency, France) were used in Essen. The primary embolic material used in Essen was Histoacryl (B. Braun, Melsungen, Germany) until 2006, when the Onyx (Medtronic, Irvine, CA, USA) began to be used. Other less frequently used techniques included platinum microcoils (EFMT, Bochum, Germany; Target Therapeutics, Fremont, CA, USA) and polyvinyl alcohol particles (Contour SE, Boston Scientific, Natick, MA, USA). Since 2007, cases treated in Stuttgart have been embolized predominantly with Glubran2 (GEM SRL, Viareggio, Italy) with standard flow-directed microcatheters (Marathon/Mirage, Medtronic; Dublin, Ireland; Magic 1.2, Balt Extrusion, Montmorency, France).

Most procedures were performed through arterial femoral access with a 6 F sheath and 6 F guide catheter, and systolic blood pressure was maintained between 120 and 140 mmHg. The main goal of embolization was to facilitate subsequent microsurgical resection by decreasing intraoperative bleeding by diminishing the AVM nidus volume; occluding as many arterial feeders as possible, particularly deep feeders; and embolizing high-rupture risk features, such as high-flow arteriovenous shunts and flow-associated aneurysms. Therefore, our procedures did not follow an intention-to-cure strategy, thus theoretically decreasing unnecessary risks associated with aggressive devascularization. The percentage devascularization was classified by the interventional neuroradiologists in charge of the procedure, who used a three-stage grading system (less than 50%, 50–80%, and >80% devascularization), after comparing the change in size and flow between the baseline and final post-procedure angiographic studies. Embolization was terminated in the case of complete embolization of the lesion, and when further embolization was deemed technically impossible or too risky. If the arteriovenous shunt of an AVM was occluded after the embolization, the patient was excluded from the study. When multiple sessions were planned, the time period in between was 6 weeks, on average.

### 2.4. Microsurgical Resection

The microsurgical procedures were performed within 1 week after the last embolization by trained vascular neurosurgeons from both centers and at referring hospitals. The surgical approach and the craniotomy were decided upon according to the location and angiographic characteristics of the lesion. Systolic blood pressure was strictly controlled and maintained in the 100–110 mm Hg range. The primary principles of this procedure were the identification of superficial vessels involved and the preservation of the main drainage veins, subarachnoid dissection, occlusion of arterial terminal afferents near the nidus, circumferential dissection of the nidus from the brain, disconnection of deep feeders, disconnection of the main drainage veins, and *en bloc* nidus removal. All AVMs were resected with a surgical microscope (e.g., Carl Zeiss, Oberkochen, Germany; Möller-Wedel, Wedel, Germany). Neurophysiological monitoring was used for AVMs located in eloquent areas. Intraoperative vascular imaging, either indocyanine green videoangiography or fluorescein videoangiography, was generally required to map the angioarchitecture of the lesion intraoperatively. Neuronavigation was considered for lesions with deep-seated components and, in general, to minimize the surgical approach. Patients underwent routine CT and DSA controls performed immediately after surgery or within 24 h. Post-embolization and post-surgical complications, resection status, and the number of operations, according to the clinical records, were analyzed.

### 2.5. Follow-Up and Outcomes

After discharge, long-term follow-up was conducted in person or via a structured telephone follow-up for all patients. The outcome “death or dependency after the last follow-up” was calculated as the percentage of patients with mRS scores ≥ 3 at the time of the last follow-up, regardless of their previous neurological status. “Complete surgical resection” was defined as the surgical resection of the lesion *en bloc*, with successful confirmation of the absence of nidus and premature filling of draining veins. For patients with a favorable status at presentation (mRS score < 3), any worsening of neurological functional status (on the basis of the mRS score), at any point in therapy, compared with the baseline mRS score, was considered “new neurological deficit” associated with treatment. In case the deterioration was still observed after discharge after at least 3 months of follow-up, and the patient’s final mRS was ≥3, the outcome was defined as “permanent disabling neurological complications after last follow-up.” If the last follow-up mRS score was <3, the outcome was considered “non-disabling neurological complications after last follow-up” [23].

### 2.6. Statistical Analysis

The statistical difference in the composition of patients between the two ruptured status groups was conducted with Fisher’s exact test for categorical dichotomic variables and χ^2^ test for categorical variables with more than two levels. The analyzed variables were demographic traits; AVM grade; nidus size; eloquence; venous drainage; presence of aneurysms; aneurysm type; presence of venous varix; AVM side and location; percentage devascularization; preoperative SRS; mRS scores before embolization, after embolization, after surgery, and at the last follow-up; as well as the shift from a benign to a negative functional neurological status (mRS score shift from <3 to ≥3) after treatment. A post hoc analysis was performed to discern in which subanalyses the statistically significant differences lay. Independent *t*-tests and one-way ANOVA with post hoc analysis were performed for continuous variables such as patient age, number of embolization sessions, and number of surgeries. Univariate and multivariate logistic regression analyses were performed to evaluate the association of predictor variables, which were then used in contingency tables, with the outcomes “death or dependency after the last follow-up (mRS score ≥ 3),” “complete surgical resection,” and “permanent disabling neurological complications.” *p* < 0.05 was considered statistically significant. All variables with significant associations in the univariate analyses were input to the multiple logistic regression model; however, the SM score was not used in this analysis because its separate components were already included in the model. Statistical analyses were performed in SPSS (version 22.0, IBM).

## 3. Results

### 3.1. Baseline Characteristics

From a total of 250 consecutive patients, 109 females (43.6%) and 141 males (56.4%) who underwent preoperative embolization and microsurgical resection were analyzed in this study. Data regarding demographic, clinical, and AVM features are described in Table 1. The mean age was 56.26 ± 16.28 years (range 10–95 years). Only one pediatric patient, a 10-year-old boy with an unruptured SM grade IV AVM who presented with epilepsy, was included in this study. From a clinical viewpoint, 87 cases (34.8%) presented with bleeding. Among these, 70, 18, and 23 cases exhibited intracerebral, subarachnoid, and intraventricular hemorrhage, respectively. Of the non-hemorrhagic cases, 73 (44.8%) presented with epilepsy, 54 (33.1%) presented with headaches, 15 (9.2%) presented with neurological deficits, and 21 (12.9%) were incidental.

A total of 150 AVMs (60.0%) were classified as SM grade III, 82 AVMs (32.8%) were classified as SM grade IV, and 18 AVMs (7.2%) were classified as SM grade V. No significant differences were observed in SM grades between ruptured and unruptured cases. The nidus size was classified as small (<3 cm), medium (3–6 cm), or large (>6 cm) in 11.7%, 73.6%, and 14.7% of the unruptured cases, respectively. In the ruptured group, the respective percentages observed were 25.3%, 62.1%, and 12.6%. A total of 127 and 62 AVMs (77.9% and 71.3%) were located in an eloquent area in the unruptured and ruptured groups, respectively.

Among unruptured AVMs, 127 cases (77.9%) had an eloquent localization, 108 cases (66.3%) had deep venous drainage, 12 cases (7.4%) were infratentorial, and one case (0.6%) was located on the midline. In contrast, the ruptured AVMs included 62 cases (71.3%) in eloquent areas, 72 cases (82.8%) with deep venous drainage, 19 (21.8%) infratentorial cases, and 9 (10.3%) midline cases. Associated aneurysms were observed in 22.8% of the entire series (16% of unruptured and 35.6% of ruptured AVMs), and a venous varix was found in 4.8% (4.3% of unruptured and 5.7% of ruptured AVMs). Among these angiographic characteristics, a small nidus (<3 cm), deep venous drainage, concomitant aneurysms, and infratentorial or midline locations were observed significantly more frequently in the ruptured than in the unruptured group (*p* = 0.021, *p* = 0.006, *p* = 0.001, *p* = 0.001, and *p* = 0.001, respectively).

The baseline unfavorable neurological functional status before treatment (mRS score ≥ 3) also significantly differed between groups and was observed in only three patients (1.8%) with unruptured AVMs but 22 patients (25.3%) with ruptured AVMs (*p* = 0.001). The rest of the independent variables analyzed between these groups were similar (Table 1).

### 3.2. Treatment and Follow-Up

All patients included in this study received preoperative embolization and subsequent surgical resection. The most common embolization agents used were Histoacryl (B. Braun, Melsungen, Germany), in 32.4% of cases; Glubran (GEM SRL, Viareggio, Italy), in 26.4% of cases; Onyx (Medtronic, Irvine, CA, USA), in 18.4% of cases; and polyvinyl alcohol particles (Contour SE, Boston Scientific, Natick, MA, USA), in 16.4% of cases. Other materials less frequently used (6.4% of cases) were Magic Glue (Balt, Montmorency, France), platinum microcoils (EFMT, Bochum, Germany; Target Therapeutics, Fremont, CA, USA), Embosphere microspheres (Merit Medical, Maastricht, The Netherlands), and detachable balloons. The average number of embolization sessions was 5.9 ± 5.68 (mean ± SD) for unruptured AVMs and 5.2 ± 5.13 for ruptured AVMs. The percentage devascularization achieved after all endovascular sessions was <50% in 24 cases (9.6%), 50–80% in 55 cases (22%), and >80% in 171 cases (68.4%). This percentage was similar between ruptured and unruptured AVMs (Table 1). Among cases with less than 50% devascularization, 54.2%, 20.8%, and 25% had AVMs classified as SM III, IV, and V, respectively. For cases with 50–80% devascularization, 56.4%, 41.8%, and 1.8% had AVMs classified as SM III, IV, and V, respectively; and for those with devascularization percentages >80%, 62%, 31.6%, and 6.4% had lesions classified as SM III, IV, and V, respectively. The comparison between these groups indicated that <50% devascularization was achieved in a significantly higher proportion of lesions classified as SM grade V, with nidus larger than 3 cm, and with infratentorial or left-side locations (*p* = 0.003, *p* = 0.004, *p* = 0.032, *p* = 0.018, respectively; Table 2). After the same baseline observation, the post-embolization proportion of patients with an unfavorable neurological status (mRS score ≥ 3) significantly differed between unruptured and ruptured AVMs (5.5% and 28.7%, respectively; *p* = 0.001). No patients died after embolization. A total of 51 patients experienced complications. Regardless, only 12 (5.3%) were considered severe (seven cases were classified as ischemic, and five cases were classified as hemorrhagic).

The average number of surgical sessions was 1.09 ± 0.28 (mean ± SD) for unruptured AVMs and 1.15 ± 0.39 for ruptured AVMs (non-significant). After surgical treatment, the proportion of patients with unfavorable neurological status (mRS score ≥ 3) was significantly higher in those with ruptured than unruptured AVMs (37.9% and 22.7%, respectively; *p* = 0.011). Four patients died after surgery (postoperative mortality rate: 1.6%). A total of 85 patients experienced complications associated with surgery. Regardless, only 36 cases (14.4%) were considered severe (19 cases were classified as ischemic, and 17 were classified as hemorrhagic).

**Figure 1:**
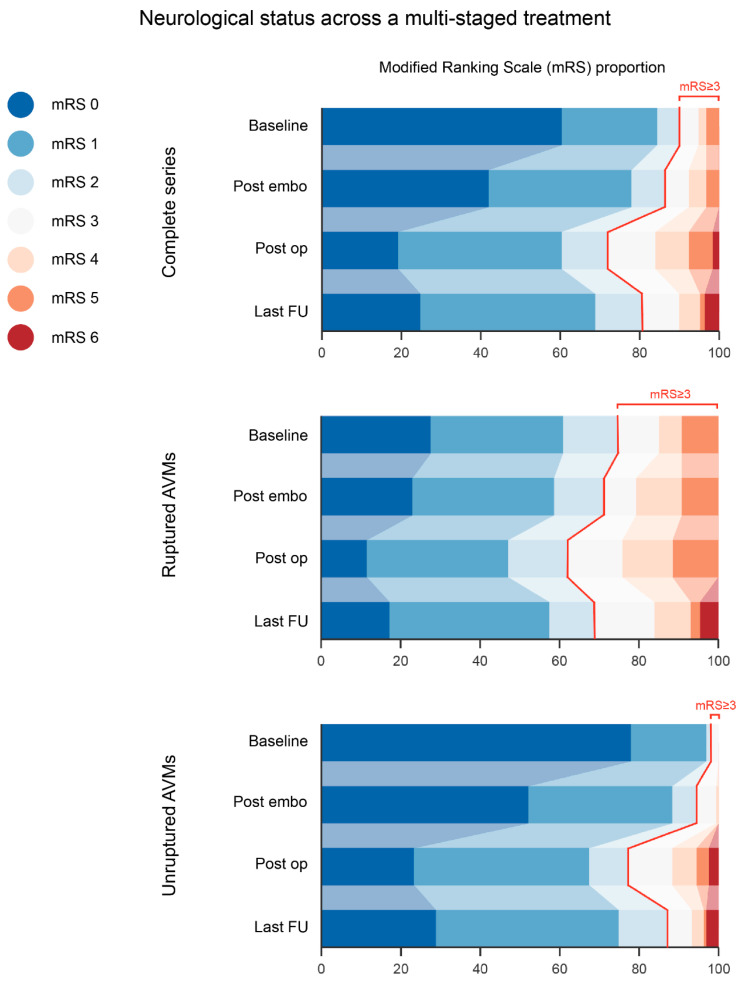
Neurological status across a multi-staged treatment. A general trend towards functional worsening after both treatment modalities, embolization (as a whole) and surgery, was evident, terminating with a noticeable improvement at the time of the last follow-up. Ruptured and unruptured cases followed the same evolution; however, the proportion of disabled patients was significantly higher at each therapy point in the ruptured subgroup. AVM: arteriovenous malformation; FU: follow-up.

Clinical and radiological follow-up was performed for all living patients. The mean follow-up time was 8.04 ± 7.86 years and was at least 3 months for 218 patients (87.2%). The resection status of the lesion was obtained for 232 of the 250 cases (92.8%). Twenty-five patients (10%) at presentation, 34 (13.6%) patients after embolization, 70 (28%) patients after surgical resection, and 48 (19.2%) patients at the last follow-up were classified as having unfavorable neurological status (mRS score ≥ 3). This tendency toward gradual functional worsening after endovascular and surgical treatment and the final improvement after the last follow-up was observed for both ruptured and unruptured AVMs (Figure 1); however, both groups showed significant differences in each analyzed period. At the last follow-up, the proportion of patients with unfavorable neurological status (mRS score ≥ 3) continued to be significantly higher for the ruptured than the unruptured group (12.9% and 31% for unruptured and ruptured AVMs, respectively; *p* = 0.001). Considering the baseline and the final neurological functional status obtained for this series, a shift from a favorable to an unfavorable mRS score (mRS score < 3 to ≥3) was observed in only 29 cases (11.6%) and was not associated with the rupture status of the lesion (Table 1). The overall mortality rate after follow-up was 3.6% (nine cases).

### 3.3. Outcomes

Death or dependency after last follow-up (mRS score ≥ 3) was observed in 48 cases (250/250, 19.2%), and complete resection was achieved in 190 patients (232/250, 82.3%). Among patients who were independent at the time of presentation (mRS score < 3), 26 (197/250, 13.2%) had permanent disabling neurological complications after at least 3 months of follow-up. Univariate analysis revealed that in the 40–59 year (*p* = 0.001) and >80 year (*p* = 0.001) age groups, mRS score ≥ 3 at presentation (*p* = 0.001), infratentorial (*p* = 0.017) or midline location (*p* = 0.003), rupture (*p* = 0.001), and presence of aneurysms (*p* = 0.001) were factors associated to death or dependency after the last follow-up (mRS score ≥ 3). Nevertheless, multivariate logistic regression analysis revealed that only age >80 years (*p* = 0.001; odds ratio, 9.398; 95% CI, 2.689–32.843) and mRS score ≥ 3 at presentation (*p* = 0.001; odds ratio, 30.376; 95% CI, 8.106–113.831) were significantly associated with this outcome (Table 3).

Complete surgical resection of the lesion was achieved in 82.3% of cases. Univariate analysis revealed that ages of 60–79 years (*p* = 0.009) and >80 years (*p* = 0.001); SM grades III (*p* = 0.001) and IV (*p* = 0.004); Spetzler-Ponce C (*p* = 0.001); nidus size >6 cm (*p* = 0.002); deep venous drainage (*p* = 0.012); rupture status (*p* = 0.013); and percentage devascularization <50 (*p* = 0.011) were factors contributing to achievement of complete surgical resection. Multivariate logistic regression analysis revealed that only age >80 years (*p* = 0.008; odds ratio, 0.255; 95% CI, 0.093–0.703), nidus size >6 cm (*p* = 0.001; odds ratio, 0.241; 95% CI, 0.103–0.565), and deep venous drainage (*p* = 0.011; odds ratio, 0.332; 95% CI, 0.142–0.779) were indeed significantly associated with this outcome. AVM grading was not included in the multivariable analysis because its separate components were already included in the model (Table 4).

The presence of any new (not registered at presentation) neurological deficit was observed in 69 cases (27.6%) after embolization. However, only 15 of those cases of deficit (6.7%) were considered disabling (mRS score ≥ 3). After surgery, a new neurological deficit was encountered in 137 cases (60.9%), only 21.8% of which were considered disabling. However, after a slight recovery trend, indicated by the progression in neurological functional status of the series, at the time of the last follow-up, acquired neurological deficit was diagnosed in 120 patients (53.3%), and was disabling in 29 patients (12.8%; Table 5). This modest recovery may reflect the partially reversible nature of surgery-related neurological deficits.

Of those 29 cases, 26 had a follow-up of at least 3 months. Univariate analysis of those 26 cases revealed that ages of 40–59 years (*p* = 0.034) or >80 years (*p* = 0.002); infratentorial location (*p* = 0.005); and presence of aneurysms (*p* = 0.015) were factors contributing to permanent disabling neurological complications after the last follow-up. Nevertheless, multivariate logistic regression analysis revealed that only age >80 years (*p* = 0.018; odds ratio, 4.061; 95% CI, 1.269–12.995) and infratentorial location (*p* = 0.005; odds ratio, 3.689; 95% CI, 1.328–10.247) were significantly associated with this outcome (Table 6). Among the complications observed after embolization and surgery, 48 cases (21.3%) were considered permanent at the last follow-up; however, only 27 cases (12%) were considered disabling (10 were classified as ischemic, and 17 were classified as hemorrhagic).

## 4. Discussion

In this study, 250 consecutive patients who presented to two German neurovascular centers with ruptured or unruptured AVMs classified as SM grade III, IV, or V were treated with a multimodal approach consisting of preoperative endovascular devascularization and subsequent microsurgical resection. Despite the complex angioarchitecture of these lesions, we were able to achieve complete resection of the lesions in 82.3% of the cases by using this modality, with an overall mortality of 3.6%. Our safety-associated outcomes indicated that 18.8% of patients experienced death or dependency (mRS score ≥ 3) after the last follow-up, and among previously independent patients after at least 3 months of follow-up, 13.2% experienced permanent disabling neurological complications.

The perioperative risks for SM grades III, IV, and V, and the subsequent efforts to further stratify and define the surgical cutoff for acceptable risk for high-risk lesions [12,13,24], led to the consensus that SM grade III (also classified as Spetzler-Ponce B) are candidates for multimodal treatment, and SM grade IV and V (Spetzler-Ponce C) may fare better when treated conservatively, with the exception of cases with recurrent bleeding and progressive neurological deficits [11]. Nonetheless, some researchers have argued that this group of high-grade AVMs has a higher future hemorrhage risk than that of AVMs as a whole [8,9]. Therefore, several other factors beyond the information indicated by the grading system should influence the decision-making process for the treatment of high-grade AVMs. Examples include the presence of demographic or angioarchitecture bleeding risk factors or a disproportionately high lifetime risk of rupture, as exhibited in younger patients. Li et al. [25] have compared conservative treatment versus intervention for 82 patients with SM grade IV and V AVMs. Although the long-term neurofunctional outcomes were similar in both groups after a 4.7-year follow-up, the interventional group exhibited advantages in the prevention of severe disability, better seizure control, and avoidance of subsequent hemorrhage. A subgroup analysis of the intervention group has demonstrated that microsurgery and hybrid surgery achieved better complete obliteration rates than embolization, and hybrid surgery resulted in less intraoperative blood loss than microsurgery alone.

The goal of AVM treatment is to eliminate the risk of hemorrhage, which is accomplished only after the complete exclusion of the lesion. Incomplete devascularization has been associated with an increase in hemorrhagic risk as high as 4 times [26]. With increasing angioarchitectural complexity, the odds of achieving a safe, complete surgical resection significantly decrease [11,12]. Likewise, the probability of complete curative obliteration with embolization alone has been reported to be below 20% [11,27], primarily because only a minority of AVMs have a sufficient proportion of pedicles that can be safely catheterized, particularly for more complex lesions. As a stand-alone modality, SRS also fails as an effective treatment for lesions with larger volumes and diameters [28,29].

This dilemma has led to attempts to combine several multimodal approaches to achieve the benefit of each individual modality while simultaneously maintaining procedural risks lower than expected from the lesion’s natural history. One example, and the strategy followed in our study, is combined multi-stage endovascular embolization followed by microsurgical resection. In this modality, preoperative embolization offers the advantage of eliminating deep arterial feeders that usually limit the resectability of large and complex AVMs. In addition, arterial inflow to the nidus is substantially decreased, thereby diminishing intraoperative blood loss and favoring an effective dissection from the surrounding cortical areas [14,15]. A progressive decrease in blood flow into the nidus by subsequent embolization sessions is believed to diminish the risk of normal perfusion pressure breakthrough, which is considered the main cause of postoperative hemorrhage in high-flow and high-volume AVMs [16,17]. This multi-stage strategy has drawbacks of additive procedural risk with each endovascular session and an elevated hemorrhagic risk because of technical complications or altered flow dynamics [30]. Another concern is the possibility of feeder reopening and recruitment in the interval between sessions; some authors have recommended intervals of no more than 3 weeks between sessions and of no more than 10 days between the last embolization and surgery [31,32]. Although comparative evidence supporting this claim remains lacking, we followed a similar schedule on the basis of physiological concepts.

Few published studies have investigated preoperative embolization in high-grade AVMs. To our knowledge, this report describes the largest cohort study to date. Del Maestro et al. have presented a case series of 27 surgically managed patients who also received preoperative embolization with Onyx 18 and have demonstrated rates of complete resection and unfavorable outcomes (mRS score 3–6) of 88.9% and 7.4%, respectively, without associated mortality. Among these patients, 88.9% had SM grade III, IV, or V AVMs. On the basis of the high proportions of complex lesions, their results indicated that multimodal management was safe and effective in their setting [32]. Another small single-center study in 13 patients with only SM grade III, IV, or V AVMs has observed similar results. After stage Onyx embolization and posterior microsurgical resection, the authors observed a 100% cure rate, defined as an absence of permeable AVM despite any Onyx remnant. The disabling and non-disabling complication rates were 15.4% and 15.4%, respectively [17].

To date, no randomized clinical study has compared the effectiveness of preoperative embolization and microsurgery against stand-alone microsurgery. A recent systematic review and meta-analysis, including 36 studies with 752 patients treated with microsurgical resection alone and 1352 patients who underwent combined preoperative embolization and resection, has not indicated any significant differences in the overall safety profile, treatment efficacy, or rate of favorable outcomes between both treatment strategies [19]. The multimodal group included 42% ruptured lesions, and only 64% of the AVMs were of SM grade III, IV, or V. This modality achieved a 96.6% complete resection rate, an overall mortality rate of 2.1%, and favorable outcomes in 90.1% of patients. In the high-grade AVM subgroup, defined as SM grade IV and V, preoperative embolization was significantly associated with a lower rate of ischemic complications, but not with postoperative hemorrhagic complications or complete resection rates. Despite the apparent lack of clear benefits with multimodal therapy compared with stand-alone surgery, importantly, none of these studies were randomized, and patients for whom a multimodal treatment was decided upon would probably have had much poorer outcomes with surgery alone. The heterogeneous nature of these lesions poses a major challenge in comparing different studies or therapy groups. That almost no significant differences have been encountered may reflect neurosurgeons selecting less-complex lesions for exclusively surgical treatment; this possibility would support the value of preoperative embolization as an adjunct therapy for complex lesions.

All our cases received embolization to facilitate subsequent surgery, and an intention-to-cure strategy was not followed, thus theoretically decreasing unnecessary risks associated with aggressive devascularization. In contrast, because most of our cases had an intermediate to large nidus (83.6%), we purposely followed step-wise devascularization to avoid the risk of normal perfusion pressure breakthrough, which can increase procedural risks [16,17]. The mean number of sessions was 5.65 (±5.5), higher than that in other multi-stage series [17,32]. Regardless of the number of sessions, we observed severe complications after embolization in 5.3% of our cases, 3.1% of which were ischemic and 2.2% of which were hemorrhagic in nature. No patients died as a result of embolization. Our endovascular results were similar to those in the combined treatment group of the most recently published meta-analysis, which has reported 3.1% and 3.4% rates of ischemic and hemorrhagic complications, respectively, and a 1.1% mortality rate after embolization; additionally, in contrast to our findings, 36% of their lesions consisted of low-grade AVMs [19].

Because our study spans an extensive time period, several embolic agents were tested as new options and evidence became available. Nevertheless, most of our procedures, including the most recent ones, were performed with cyanoacrylates, either Histoacryl (32.4%) or Glubran (26.4%), followed by copolymers such as Onyx (18.4%). A meta-analysis comparing cyanoacrylates with Onyx has found that cyanoacrylates are associated with lower permanent complication rates, whereas Onyx has higher angiographic cure rates [33]. We believe that the embolic properties of cyanoacrylates are particularly favorable for preoperative procedures when the main objective is the occlusion of high-flow shunts, intranidal aneurysms, and deep feeders, rather than the complete embolization of the lesion. Price and availability are also advantages supporting the use of these agents. Moreover, Onyx has been associated with intraoperative sparking and combustion when bipolar cautery is applied close to an embolized nidus [34], a necessary step for microsurgical resection. The above-described meta-analysis has confirmed that, among multimodal approaches, cyanoacrylates have been used by most studies [19].

Interestingly, despite the complexity of our cohort and the goals of embolization, most patients achieved devascularization percentages of >80% (68.4% of our cases), and 90.4% achieved at least 50% occlusion. These findings are very similar to the average results of the prior meta-analysis [19], in which 90.6% of patients had at least a 50% AVM volume decrease, even though some included cohorts were treated with an intention-to-cure strategy. We observed a trend toward incomplete surgical resection after lower percentages of devascularization (<50%); however, this association was significant only in the univariate analysis. Furthermore, higher devascularization percentages were not associated with our two other safety outcomes: death or dependency and disabling neurological complications after the last follow-up. In a matched series, including single-stage and multi-stage treatment, Zeng et al. have also not found a significant association between embolization degree (<30%, 30–60%, and >60%) and clinical outcomes [35]. In light of our results, although statistical significance was lacking, we believe that achieving greater percentages of devascularization may benefit successful treatment without additional complications.

We were able to achieve complete surgical resection in 82.3% of our cases, with surgery-associated and overall mortality rates of 1.6% and 3.6%, respectively, values slightly inferior to the 96.6% rate of complete resection and 2.1% mortality obtained in the pooled analysis by Park et al. However, their proportion of complex cases was clearly lower because only 64% of their AVMs were classified as SM III or above, and only three retrospective studies among the 36 studies included encompassed these types of lesions exclusively [19]. Among these examples, in the previously largest single-center case series, comprising 67 patients treated with this modality, a complete resection rate of 94% and a 10% mortality rate were demonstrated. Interestingly, 26.7% of their diagnosed high-risk AVMs were excluded from treatment with this modality because, according to the authors, those patients were intended to be managed non-surgically [20]. As with most other series, the criteria used to determine surgical feasibility in this cohort is unclear.

More recently, the experience at a tertiary neurovascular center using a hybrid intention-to-treat, single-stage treatment for SM grade III, IV, and V AVMs has been published. The rationale for preferring a single-stage paradigm, according to the authors, was to minimize radiation exposure, procedural morbidity and mortality, the inter-procedural rupture rate, and patient anxiety. Complete surgical resection was obtained in 96.8% of patients, and the mortality rate reached 3.2%. These remarkable results may be explained partly by the treated subgroups representing only a fraction of the diagnosed patients (36.4%) with these gradings and by 80.6% of treated cases having grade III AVMs [18]. This hybrid single-stage approach has been described as a feasible alternative in the treatment of complex AVMs, thus enabling complete treatment to be performed in the same session by using a hybrid operating room [35,36]. Beyond complete surgical resection, other outcomes reflecting surgical performance for AVM surgery have been proposed, such as operative time, operative blood loss, and clip usage [4]; however, their relationships with preoperative embolization have been inconsistent [18,35,37]. We were unable to explore the effects of preoperative embolization on these variables because of the limitations of the data set.

Our registries indicated that between January 1989 and February 2023, a total of 1253 patients were diagnosed with AVMs, and 422 AVMs were classified as high-grade (SM grade III, IV, and V). The 250 consecutive patients treated under this paradigm represent 59.2% of this group, besides the 4.8% who were considered beyond the limits of treatment. This proportion of multimodality-eligible patients is higher than that in previous studies; however, we were unable to establish which variables influenced the treatment criteria, on the basis of the current literature. Our mean follow-up of more than 8 years is one of the longest among similar publications, and 87.2% of our patients had at least 3 months of follow-up after hospital discharge. From a safety perspective, we found an 18.8% rate of death or dependency (mRS score ≥ 3) after the last follow-up for all patients, regardless of their admission neurological status. This aspect is important because we included 34.8% of ruptured cases, and 10% of our admitted patients had mRS scores ≥3 at presentation. After multivariate analysis, only two variables were identified as significant predictors of this outcome: age >80 years (*p* = 0.001; OR 9.398), as further discussed below, and mRS score ≥ 3 at baseline (*p* = 0.001; OR 30.376). As described by Hartmann et al., many publications have not provided the patient’s baseline functional status or information comparing the functional changes between different therapies [38]. A general trend toward functional worsening after both treatment modalities, embolization (as a whole) and surgery, was evident, but a noticeable improvement was ultimately found at the time of the last follow-up (Figure 1). The effects of each therapy on functional neurological status have been previously described [11], and the series’ final recovery reflects the partially reversible nature of surgery-associated neurological deficits [15,23,38]. This progression was followed by ruptured and unruptured cases; however, the proportion of disabled cases was significantly higher at each point of therapy in the ruptured subgroup.

We also considered that differences among mRS scores throughout the course of the therapy might be a more sensitive method to diagnose neurological deficits than simply searching for events listed as neurological deficits or complications in patient registries. We searched for any functional worsening at any point during therapy; if the end result was an mRS score ≥ 3, the deficit was considered disabling [23]. As previously discussed, the odds of a death or disability outcome were 30 times higher for those who presented with an mRS score ≥ 3 before treatment. To elucidate additional factors that might affect a patient’s prognosis, we investigated neurological complications after excluding previously disabled patients. Considering the potentially reversive nature of postoperative deficit, we decided to analyze only patients with at least 3 months of follow-up. Among this subgroup, we observed a rate of permanent disabling neurological complications of 13.2%. Prior case series composed exclusively of SM grade III, IV, or V AVMs have indicated unfavorable functional outcome rates ranging between 3.2% and 19.4% [17,18,20]. Our results are within the previously described range.

We believe that the differences between our data and previous studies highlight a generalized lack of standardization in patient selection, treatment protocols, and study outcomes. Until prospective and randomized studies are published, these factors will continue to hinder more precise conclusions from being drawn. In contrast, we emphasize that the high angioarchitectural heterogeneity in these high-grade lesions, coupled with their low incidence, may preclude the formulation of future recommendations without substantial bias. However, the post-ARUBA reaction and consequences [6] should serve as a cautionary tale of how incomplete evidence can influence clinical practice and, most importantly, patient outcomes. In the meantime, we consider that the optimal decision to treat should be determined by multidisciplinary teams on a case-to-case basis by consideration of lesion bleeding risk (discussed below), treatment-associated risk factors, angioarchitectural features, life expectancy, and patient preference.

### 4.1. Risk Factors

Several demographic, morphological, and angioarchitectural risk factors for hemorrhage have been described; nonetheless, these findings often differ among studies and therefore should be interpreted with caution [7]. Among other factors, previous hemorrhage [1,39], small nidus size [40,41,42,43], deep venous drainage [1,44], impaired venous drainage [45,46], single vein drainage [47], deep brain location [1,48], infratentorial location [49], periventricular location [50], associated aneurysms [50,51,52], older age [1,53,54], female sex [55], and hypertension [44], have been associated with higher bleeding risk.

Small nidus size has been associated with hemorrhagic presentation in several studies [40,41,42,43], although not with bleeding after the first episode [56]. Spetzler et al. [57] have intraoperatively recorded the arterial pressure of the main feeding vessels of 24 AVMs, and have determined that smaller AVMs have significantly higher feeding artery pressures than larger AVMs, and are also associated with larger hemorrhage volumes. The higher the resistance as a consequence of a smaller nidus, the higher the perfusion pressure inside the AVM; this relationship may physiologically determine whether rupture occurs and have an influence on the size of the resulting hematoma [58,59]. In this case series, we found a significantly higher proportion of lesions with a small nidus (<3 cm) in the ruptured than in the unruptured group (*p* < 0.021), similarly to previously published data. Our analysis did not include ICH size because this information was not available in the earliest records. However, we did include other types of bleeding patterns, such as SAH and IVH, which are usually excluded in investigations of this relationship as a source for potential bias [56]. Nidus size was associated with both endovascular and microsurgical treatments. A high proportion of large nidus AVMs (>6 cm) had less than 50% endovascular devascularization (*p* = 0.004) and were negatively associated with the odds of achieving complete microsurgical resection (*p* = 0.001; OR 0.241), although no repercussions in terms of safety outcomes were observed. This reflects a higher technical difficulty as well as our devascularization goal, which did not include the occlusion of superficial or peripheral portions not supposed to restrict surgical resection.

We observed a significantly higher proportion of deep venous drainage among patients who presented with hemorrhage (82.8% vs. 66.3% for unruptured AVMs, *p* = 0.006). This anatomical feature was also significantly and negatively associated with complete microsurgical resections in the multivariate analysis (*p* = 0.011; OR 0.332). For the purpose of this study, we considered the presence of any deep venous drainage, which has been associated with an elevated risk of microsurgical resection [60]. In contrast, exclusively deep venous drainage has been associated with an elevated risk of spontaneous hemorrhage in the natural course of the lesion [46]. Because the data originated from different sources and some data were from more than a decade ago, we were unable to determine which cases met this definition. Nonetheless, we can infer that a substantial proportion of these lesions might have had exclusively deep drainage, given their observed significant relationship with rupture status.

AVMs in the infratentorial compartment represent approximately 15–20% of brain AVMs, and this location is independently associated with hemorrhagic presentation [49]. Although this study was a tertiary center series, we observed a lower-than-expected proportion of infratentorial lesions (11%), yet these lesions constituted a substantial proportion of ruptured cases (21.8% vs. 7.4% of unruptured AVMs, *p* = 0.001). We also found that this location was a risk factor for treatment-associated permanent disabling neurological deficits (*p* = 0.012; OR 3.689), although it was not found to be significant after the multivariate analysis for death or dependency after the last follow-up outcome. This finding can be explained by anatomy itself. The lower endocranial volume of the posterior fossa results in a decrease in compliance in the event of hemorrhage or edema, whether associated with presentation or treatment factors. In contrast, the tightly packed nuclei and tracts inside the brainstem and narrow surgical corridors surrounded by critical structures can increase the risks associated with surgery and endovascular therapy. Morbidity and mortality rates for posterior fossa AVMs treated surgically range between 13% and 25%, and 7% and 15%, respectively [61,62,63]. Unlike previous studies that have reached the conclusion that the fate of infratentorial AVMs is determined by their overall worse presurgical condition [64], in our study, because this location was only found to be significant with functional outcomes when ruptured and disabled cases were excluded from the analysis, we concluded that it was indeed a risk factor associated with treatment. The inclusion of three non-cerebellar lesions (9.7% of the infratentorial subgroup), in contrast to the aforementioned study, and the grading score of at least III in all our cases, resulted in more complex lesions subject to interventional complications.

Advancing age has been described as an independent predictor of hemorrhagic presentation [54] and high-risk angioarchitectural features, such as small nidus, venous ectasia, and feeding artery aneurysms [65]. The negative effect of age on surgical outcomes is emphasized by its incorporation as a factor in the supplementary SM grading system, wherein patients older than 40 years of age have 3 points added to their score, thus potentially resulting in falling beyond the limits of operability, as the authors propose [13]. Older patients have a limited capacity for neurological rehabilitation and higher perioperative risks than younger patients—a trend observed in other neurosurgical pathologies [66]. However, because patients currently benefit from a prolonged life expectancy, better policies for disease prevention, and lower perioperative risk factors, some authors have advocated for AVM microsurgery among selected older patients with generally favorable outcomes [67]. Our institutional policy follows this philosophy: age in itself was not considered an exclusion criterion for treatment. The largest cohort of surgically treated patients older than 65 years demonstrated overall favorable outcomes, defined as an mRS score of 0–3, in 71% of cases, and no difference was observed for the subgroup above 70 years of age [68]. In contrast to our series, why the favorable outcome included patients with mRS scores of 3, who usually are considered disabled, is unclear. The aforementioned series had 63% of AVMs with SM grade I and II, which have traditionally been associated with better surgical results; therefore, comparisons with the present data are conflicting. In our series, the group of patients >80 years of age who received multimodal treatment consisted of 22 cases (8.8%). A significantly higher proportion of patients in this age group were observed among ruptured AVMs (17.2% vs. 4.3% for unruptured cases, *p* = 0.006), according to previous evidence, and was a significant predictor for unfavorable safety and effectiveness outcomes in the respective multivariate analyses. Being in this group was associated with death and dependency at the last follow-up (*p* = 0.001; OR 9.398) and with permanent disabling neurological deficit at the last follow-up (*p* = 0.018; OR 4.061), and was negatively associated with complete microsurgical resection (*p* = 0.008; OR 0.255). On the basis of these results, we conclude that, even with all benefits of modern medicine, the combination of perioperative risks associated with complex AVMs and extreme age may indeed fall beyond surgical limits.

The presence of intracranial aneurysms may increase the risk of rupture when associated with AVMs [11,52,69]; however, some controversy exists regarding classification schemes, specific aneurysm-type associated risk, and when these lesions should be treated [70]. Some authors consider that flow-associated aneurysms, those arising from arteries that play a role in the perfusion of the nidus, are most likely to present with hemorrhage than intranidal aneurysms [71], and others have stated that distal flow-associated and intranidal aneurysms immediately adjacent to the site of the arteriovenous shunt may be more prone to rupture [72]. Histological evidence supports that intranidal aneurysms have more in common with the venous than the arterial system and have thinner walls [72]. In our series, we considered only flow-associated and intranidal aneurysms as “associated aneurysms,” and found that these aneurysms were present in 22.8% of cases, in agreement with previous data [70]. Interestingly, they were found in a higher proportion of the ruptured than the unruptured group (*p* = 0.001), thus supporting their roles as weak points that might precede rupture. We also found that the presence of aneurysms was predictive of the outcomes “death and dependency at last follow-up” (*p* = 0.001; OR 3.586) and “permanent disabling neurological complications” (*p* = 0.015; OR 2.838); however, this association was found only in the univariate analysis but not the multivariate analysis.

### 4.2. Limitations

Our study has several potential limitations that should be noted. First, the retrospective nature of this study in two tertiary centers and the intrinsic complexity of these lesions inevitably resulted in selection bias. Even though this is the largest reported case series of high-grade AVMs treated with multi-stage preoperative embolization and surgery, our sample size was limited, and the comparison of our results with previously published series is difficult because of high heterogeneity in treatment protocols, inclusion criteria, and outcome definitions. Therefore, our conclusions should not be generalized to an unselected population.

We were unable to compare this cohort against a pure surgical arm, and we do not have information on patients managed conservatively, which might have indicated interesting conclusions regarding multimodal treatment safety and effectiveness. We were unable to explore the role of this strategy on other surgical performance outcomes beyond complete surgical resection, owing to the limitations of our data set. Because our study spans three decades, some angioarchitectural variables were impossible to extract, in some cases because their importance was not fully understood at the time or simply because of the limitations of our data set. Finally, the use of the mRS score instead of an assessment by neurologists may underestimate complications, particularly those associated with higher cortical functions.

## 5. Conclusions

To our knowledge, this is the largest study on high-grade AVM treatment under this strategy to date. Multi-stage preoperative embolization and subsequent microsurgical resection are feasible for selected patients with high hemorrhagic risk, even for those with AVMs that are usually considered beyond surgical limits. We believe that our study makes an important contribution to the understanding of this particular subgroup of patients, particularly because only a few small studies have been published, and elucidating which subgroups of patients benefit the most from treatment and which patients should be managed conservatively is crucial. This strategy was effective overall, achieving an 82.3% rate of complete surgical resection. Preoperative embolization facilitated resection by the occlusion of deep arterial feeders and decreasing intranidal flow. A non-significant trend toward incomplete surgical resection was observed with lower percentages of devascularization (<50%); furthermore, higher devascularization percentages were not associated with negative neurological repercussions. In terms of safety, we identified an 18.8% rate of death and dependency at the last follow-up, regardless of the admission condition of the patient; moreover, 13.2% of initially independent patients were diagnosed with permanent disabling neurological complications after at least 3 months of follow-up. Treatment for AVMs with a large nidus, infratentorial location, or deep venous drainage should be decided carefully on a case-by-case basis. Age >80 years was associated with poor safety and effectiveness outcomes, thus leading us to believe that, for this patient group, treatment should be offered only in exceptional circumstances.

## Figures and Tables

**Table 1:** Characteristics of 250 patients: comparison of ruptured and unruptured AVMs.

Characteristics	Unruptured AVMs	Ruptured AVMs	*p* Value
Number of patients	163/250 (65.2)	87/250 (34.8)	
Age in years ^‡^	55.12 (14.43)	58.49 (19.15)	0.001 *
Age (years)			0.006 *
0–19	1(0.6)	0	
20–39	22 (13.5)	16 (18.4)	
40–59	77 (47.2)	31 (35.6)	
60–79	56 (34.4)	25 (28.7)	
≥80	7 (4.3) ^§^	15 (17.2) ^§^	
Sex			0.803
Male	91 (55.8)	50 (57.5)	
Female	72 (44.2)	37 (42.5)	
AVM grade			
SM III (Ponce B)	95 (58.2)	55 (63.2)	0.743
SM IV	56 (34.4)	26 (29.9)	
SM V	12 (7.4)	6 (6.9)	
Ponce C	68 (41.7)	32 (36.8)	0.448
Nidus size (cm)			0.021 *
<3	19 (11.7) ^§^	22 (25.3) ^§^	
3–6	120 (73.6)	54 (62.1)	
>6	24 (14.7)	11 (12.6)	
Eloquent area	127 (77.9)	62 (71.3)	0.244
Deep venous drainage	108 (66.3)	72 (82.8)	0.006 *
Location			
Supratentorial	151 (92.6)	68 (78.2)	0.001 *
Infratentorial	12 (7.4)	19 (21.8)	
AVM side			0.001 *
Right	81 (49.7)	38 (43.7)	
Left	81 (49.7)	40 (46)	
Midline	1 (0.6) ^§^	9 (10.3) ^§^	
Presence of aneurysms	26 (16)	31 (35.6)	0.001 *
Aneurysm type			0.661
Flow-related arterial	25 (96.2)	29 (93.5)	
Intranidal	1 (3.8)	2 (6.5)	
Presence of venous varix	7 (4.3)	5 (5.7)	0.410
mRS score ≥ 3 at presentation	3 (1.8)	22 (25.3)	0.001 *
Number of embolizations ^‡^	5.9 (5.684)	5.2 (5.133)	0.338
Percentage devascularization			0.09
<50	11 (6.7)	13 (14.9)	
50–80	39 (23.9)	16 (18.4)	
>80	113 (69.4)	58 (66.7)	
mRS score ≥ 3 after embolization	9 (5.5)	25 (28.7)	0.001 *
Number of surgeries ^‡^	1.09 (0.281)	1.15 (0.390)	0.181
mRS score ≥ 3 after surgery	37 (22.7)	33 (37.9)	0.011 *
Total resection	132 (81)	58 (66.7)	0.012 *
Preoperative SRS	16 (9.8)	10 (11.5)	0.679
mRS score ≥ 3 at last follow-up	21 (12.9)	27 (31)	0.001 *
mRS score shift from ≤2 to ≥3	18 (11)	11 (12.6)	0.707

Values are numbers of patients (%). ^‡^ Values are means (standard deviations). * Statistical significance (*p* < 0.05). ^§^ Statistically significant values after post hoc analysis.

**Table 2:** Comparison between patient characteristics and percentage devascularization after embolization.

	Percentage Devascularization			
Characteristics	<50%	50–80%	>80%	*p* Value
Number of patients	24/250 (9.6)	55/250 (22)	171/250 (68.4)	
AVM grade				0.003 *
III (Ponce B)	13 (54.2)	31 (56.4)	106 (62)	
IV	5 (20.8)	23 (41.8)	54 (31.6)	
V	6 (25) ^§^	1 (1.8)	11 (6.4)	
Ponce C	11(45.8%)	24 (43.6%)	65 (38%)	0.63
Nidus size (cm)				0.004 *
<3	6 (25)	4 (7.2)	31 (18.1)	
3–6	10 (41.7) ^§^	42 (76.4)	122 (71.4)	
>6	8 (33.3) ^§^	9 (16.4)	18 (10.5)	
Brain eloquent area	20 (83.3)	42 (76.4)	127 (74.3)	0.619
Deep venous drainage	19 (79.2)	33 (60)	128 (74.9)	0.073
AVM status				0.09
Unruptured	11 (45.8)	39 (70.9)	113 (66.1)	
Ruptured	13 (54.2)	16 (29.1)	58 (33.9)	
Location				0.032 *
Supratentorial	17 (70.8) ^§^	49 (89.1)	153 (89.5)	
Infratentorial	7 (29.2) ^§^	6 (10.9)	18(10.5)	
AVM side				0.018 *
Right	16 (66.7)	22 (40)	81 (47.4)	
Left	5 (20.8) ^§^	31 (56.4)	85 (49.7)	
Midline	3 (12.5)	2 (3.6)	5 (2.9)	
Presence of aneurysms	7 (29.2)	11 (20)	39(22.8)	0.671
Presence of venous varix	4 (16.7) ^§^	4 (7.3)	4(2.3) ^§^	0.006 *

Values are numbers of patients (%). * Statistical significance (*p* < 0.05). ^§^ Statistically significant values after post hoc analysis.

**Table 3:** Risk factors associated with death or dependency after the last follow-up (mRS score ≥ 3).

Logistic Regressions	Univariate			Multivariate		
Predictors	OR	95% CI	*p* Value	OR	95% CI	*p* Value
Age (years)						
0–19	-	-	-			
20–39	0.449	0.151–1.333	0.149			
40–59	0.279	0.132–0.591	0.001 *	0.654	0.264–1.625	0.361
60–79	1.651	0.864–3.155	0.129			
≥80	9.985	3.893–25.613	0.001 *	9.398	2.689–32.843	0.001 *
Female sex	0.907	0.479–1.716	0.764			
mRS score ≥ 3 at presentation	21.402	7.895–58.017	0.001 *	30.376	8.106–113.831	0.001 *
Location						
Supratentorial	0.370	0.163–0.836	0.017 *	0.404	0.116–1.414	0.156
Infratentorial	2.705	1.196–6.119	0.017 *	2.473	0.707–8.644	0.156
Side						
Left	0.577	0.302–1.100	0.095			
Right	1.126	0.600–2.114	0.711			
Midline	7.071	1.911–26.162	0.003 *	4.678	0.711–30.759	0.108
AVM grade						
SM III (Ponce B)	0.743	0.394–1.402	0.360			
SM IV	1.446	0.754–2.771	0.267			
SM V	0.831	0.231–2.994	0.777			
Ponce C	1.345	0.713–2.537	0.360			
Nidus size (cm)						
<3	0.682	0.269–1.727	0.419			
3–6	1.076	0.539–2.145	0.836			
>6	1.296	0.548–3.065	0.554			
Eloquent brain area	0.961	0.464–1.990	0.914			
Deep venous drainage	1.058	0.522–2.146	0.875			
Ruptured AVM	3.043	1.596–5.801	0.001 *	0.498	0.176–1.409	0.189
Presence of aneurysms	3.586	1.827–7.041	0.001 *	2.028	0.846–4.862	0.113
Presence of venous varix	1.430	0.372–5.494	0.603			
Percentage devascularization						
<50	1.460	0.547–3.902	0.450			
50–80	1.068	0.504–2.263	0.865			
>80	0.808	0.416–1.567	0.527			
Preoperative SRS	0.744	0.244–2.269	0.603			

* Statistical significance (*p* < 0.05).

**Table 4:** Risk factors associated with complete surgical resection.

Logistic Regressions	Univariate			Multivariate		
Predictors	OR	95% CI	*p* Value	OR	95% CI	*p* Value
Age (years)						
0–19	-					
20–39	0.634	0.298–1.350	0.238			
40–59	1.086	0.603–1.955	0.783			
60–79	2.598	1.268–5.324	0.009 *	2.155	0.986–4.708	0.054
≥80	0.222	0.091–0.545	0.001 *	0.255	0.093–0.703	0.008 *
Female sex	0.928	0.517–1.664	0.802			
mRS score ≥ 3 at presentation	0.429	0.181–1.012	0.053			
Location						
Supratentorial	0.716	0.281–1.826	0.485			
Infratentorial	1.396	0.548–3.561	0.485			
Side						
Left	1.430	0.795–2.571	0.232			
Right	0.677	0.378–1.212	0.189			
Midline	0.784	0.162–3.799	0.763			
AVM grade						
SM III (Ponce B)	2.953	1.624–5.369	0.001 *	-	-	-
SM IV	0.414	0.228–0.752	0.004 *	-	-	-
SM V	0.465	0.172–1.260	0.132	-	-	-
Ponce C	0.390	0.223–0.682	0.001 *	-	-	-
Nidus size (cm)						
<3	1.650	0.691–3.943	0.260			
3–6	1.607	0.874–2.955	0.127			
>6	0.306	0.145–0.642	0.002 *	0.241	0.103–0.565	0.001 *
Eloquent brain area	1.465	0.766–2.802	0.248			
Deep venous drainage	0.373	0.173–0.807	0.012 *	0.332	0.142–0.779	0.011 *
Ruptured AVM	0.470	0.260–0.850	0.013 *	0.680	0.345–1.342	0.266
Presence of aneurysms	0.851	0.432–1.676	0.641			
Presence of venous varix	0.945	0.247–3.608	0.934			
Percentage devascularization						
<50	0.327	0.138–0.776	0.011 *	0.438	0.165–1.165	0.098
50–80	1.552	0.728–3.308	0.255			
>80	1.225	0.663–2.264	0.516			
Preoperative SRS	1.367	0.492–3.797	0.549			

* Statistical significance (*p* < 0.05).

**Table 5:** Frequency of new treatment-related neurological deficits in patients with good neurological status at presentation (n = 225).

Deficit	Post-Embolization	Post-Surgery	Last Follow-Up
Disabling deficit mRS score ≥ 3	15 (6.7)	49 (21.8)	29 (12.8)
Non-disabling deficit mRS score ≤2	54 (24)	88 (39.1)	91 (40.4)
Total	69 (27.6)	137 (60.9)	120 (53.3)

Values are numbers of patients (%).

**Table 6:** Risk factors associated with permanent disabling neurological complications after at least 3 months of follow-up (26 patients).

Logistic Regressions	Univariate			Multivariate		
Predictors	OR	95% CI	*p* Value	OR	95% CI	*p* Value
Age (years)						
0–19	-	-	-			
20–39	0.202	0.027–1.539	0.123			
40–59	0.359	0.139–0.927	0.034 *	0.597	0.211–1.685	0.330
60–79	1.925	0.847–4.379	0.118			
≥80	5.133	1.865–14.131	0.002 *	4.061	1.269–12.995	0.018 *
Female sex	0.789	0.343–1.815	0.577			
mRS score ≥ 3 at presentation	-	-	-			
Location						
Supratentorial	0.257	0.101–0.658	0.005 *	0.271	0.098–0.753	0.012 *
Infratentorial	3.884	1.520–9.923	0.005 *	3.689	1.328–10.247	0.012 *
Side						
Left	0.437	0.182–1.045	0.063			
Right	1.573	0.692–3.577	0.279			
Midline	4.043	0.978–16.714	0.054			
AVM grade						
SM III (Ponce B)	0.448	0.197–1.022	0.056			
SM IV	2.246	0.990–5.097	0.053			
SM V	1.083	0.235–5.000	0.918			
Ponce C	2.230	0.979–5.081	0.056			
Nidus size (cm)						
<3	0.184	0.024–1.398	0.102			
3–6	1.515	0.583–3.938	0.394			
>6	1.540	0.540–4.393	0.420			
Eloquent brain area	1.085	0.415–2.838	0.868			
Deep venous drainage	1.062	0.426–2.650	0.897			
Ruptured AVM	1.193	0.517–2.755	0.679			
Presence of aneurysms	2.838	1.222–6.592	0.015 *	1.728	0.683–4.369	0.248
Presence of venous varix	0.775	0.096–6.254	0.811			
Percentage devascularization						
<50	0.350	0.045–2.701	0.314			
50–80	0.829	0.297–2.309	0.719			
>80	1.611	0.621–4.184	0.327			
Preoperative SRS	0.318	0.041–2.453	0.272			

* Statistical significance (*p* < 0.05).

## Data Availability

The data presented in this study are available on request from the first author. The data are not publicly available due to patient privacy protection.
